# Pollination Biology: From Pollinators and Floral Traits to Landscape Management

**DOI:** 10.3390/biology14101420

**Published:** 2025-10-15

**Authors:** Yu Gao

**Affiliations:** 1 College of Plant Protection, Jilin Agricultural University, Changchun 130118, China; gaothrips@jlau.edu.cn; 2 Key Laboratory of Soybean Disease and Pest Control, Ministry of Agriculture and Rural Affairs, Changchun 130118, China

Pollination biology examines the mechanisms, agents, and ecological significance of pollination [1,2]. As key ecosystem actors, pollinators provide vital ecological services and help maintain the dynamic balance and stability of natural systems. They are essential for the reproduction of many plant species and the production of fruits and seeds [3,4]. Their health is tightly linked to green agriculture, food security, and human nutrition. Insect pollinators alone are responsible for 80–85% of all animal-mediated pollination, a service widely recognized by both scientists and the public [5,6]. Against this backdrop, the nine papers (eight articles and one editorial) assembled in this Special Issue on *Pollination Biology* weave compelling research findings that spans scales and disciplines. Together, these studies deepen our understanding of pollination processes and offer insights into how this knowledge can inform practical, pollinator-friendly management strategies.

Contribution 1: Huancas et al. deliver the first comprehensive review of mathematical models for pollinator dynamics [7]. Their bibliometric map highlights four dominant toolkits—ODEs, PDEs, network-patch frameworks, and emerging stochastic or fractional-order models—while at the same time identifying gaps in optimal control and stochastic approaches. The findings highlight modeling trends, ecological insights, and policy applications in agriculture and conservation. This study provides a comprehensive typology of mathematical models for pollinator dynamics, bridging theoretical ecology and real-world decision-making.

Contribution 2: Layek et al. investigate seasonal shifts in floral traits, pollinator assemblages, and fruit set of the wild *Solanum sisymbriifolium* across the Rarh plains of West Bengal [8]. Their year-round monitoring reveals that although hot-season flowers are smaller and produce less nectar, large-bodied bees dominate during this period and drive the highest reproductive success. This research uniquely links microclimate-driven floral plasticity to real-time pollinator efficiency.

Contribution 3: Ejaz et al. test whether adding managed honey bee hives can improve seed yields in Punjab alfalfa fields [9]. Across three commercial farms, they trial 0–4 hives per hectare, finding a linear increase in seed yield, peaking at 38% boost with four hives. Notably, wild pollinators contributed less than 12% of total visits. The study highlights the practical value of hive supplementation as a scalable strategy to enhance forage crop profitability in Pakistan.

Contribution 4: Laboisse et al. chart how honey bees exploit floral resources across space and time in the Banja Luka region of Bosnia [10]. Using RFID-tagged workers and hive scales for two years, they show that 90% of foraging stays within 1.2 km of the hive. Bees flexibly shift their daily activity to match the phenology of sequentially blooming crops like apple, acacia, sunflower, and heather. This high-resolution calendar provides valuable guidance for synchronizing hive placement with peak nectar flows.

Contribution 5: Bukhari et al. examine the impact of nitrophos fertilizer on floral traits and pollinator visitation in Pakistan’s onion seed fields [11]. While higher fertilizer doses increase flower size, they simultaneously dilute nectar sugar concentration, reducing visits from wild bees and hoverflies by 22%, and ultimately lowering seed yield by 14% compared to moderate fertilization. This study uniquely reveals that excessive nitrogen input can disrupt plant–pollinator synergy.

Contribution 6: Layek et al. explore how seasonal changes in temperature, humidity, and photoperiod influence the floral biology and pollination of *Turnera ulmifolia* in India [12]. Long, humid days enhance pollen viability and bee visits, boosting fruit set. However, nectar thickens above 35 °C, deterring hoverflies and leading to a summer dip in yields. This work illustrates how climate-driven shifts can desynchronize plant–pollinator interaction.

Contribution 7: Zhang et al. investigate the pollination biology of *Iris setosa*, a cold-hardy ornamental native to Jilin, China [13]. Combining field observations, hand-pollination experiments, and floral scent analysis, they show that large violet perianths attract bumblebees via visual contrast, while eugenol-rich fragrances reinforce this attraction. The species is largely self-incompatible; hive supplementation raises seed set from 18% to 62%, pointing to managed bumblebees as a practical tool for commercial seed production of this northern iris.

Contribution 8: Eggers et al. identify a recessive nuclear gene (al) on the short arm of chromosome 6 in potato that causes antherless cytoplasmic male sterility (CMS) when interacting with P-type cytoplasm, but remains fertile with A- or T-type cytoplasm [14]. Exploiting this CMS system eliminates the need for manual emasculation of female parents, improves hybrid seed production, and significantly reduces berry set in F_1_ hybrids under field conditions. This work presents a valuable tool for diploid hybrid potato breeding.

Contribution 9: Leung & Reid examine Canada’s regulatory landscape for the import and movement of managed bees, focusing on risks to native pollinators in Yukon [15]. They highlight the lack of ecological risk assessments and permitting requirements in the territory. Their proposed Yukon-specific framework—combining seasonal transport limits, pathogen screening, and incentives for domestic bumblebee rearing—offers a replicable model for northern regions aiming to balance agricultural needs with pollinator conservation.

This Special Issue offers, at best, a modest snapshot of an extraordinarily complex and rapidly evolving field. While the breadth of topics covered and the depth of individual studies reflect the momentum in pollination biology, we are acutely aware that any Special Issue can only scratch the surface. These contributions are intended not as final answers, but as conversation starters. They remind us that integrative, interdisciplinary collaboration is not a luxury but a necessity if we are to deepen our still-fragmentary understanding of these remarkable organisms and craft management strategies humble enough to accommodate nature’s surprises. Pollination research remains stubbornly place-based; a shift in bloom phenology, a turnover in pollinator guilds, or even a subtle change in slope aspect can overturn yesterday’s ‘universal’ finding tomorrow. These geographic fingerprints limit how far any one dataset can travel, but they also provide the calibration marks that global models so desperately need. By treating regional idiosyncrasies as adjustable parameters rather than immovable barriers—and by openly sharing both our successes and our missteps—we aim to advance the discipline toward more robust and generalizable insights. Pollination research must move beyond simply mapping ‘who pollinates whom’. At the molecular, individual, and population levels, we need to understand how environmental factors, chemical signals, morphological fit, and behavioral decisions collectively influence pollen transfer. These mechanisms must then be translated into parameters for larger-scale models. Finally, through collaboration with agronomists, plant breeders, land managers, and policy-makers, we must translate these forecasts into actionable spatial plans, cropping systems, seed technologies, and transnational policy frameworks.

## Data Availability

The original contributions presented in this study are included in the list of contributions. Further inquiries can be directed to the corresponding author.
